# Salt forms of a thio­amide: protonation of 1-(2,6-di­methyl­phen­yl)thio­urea

**DOI:** 10.1107/S2053229626001804

**Published:** 2026-02-25

**Authors:** Kristin Donnachie, Bethany Gibson, Alan R. Kennedy, Connor MacCall, Marc Reid

**Affiliations:** a Department of Pure & Applied Chemistry, University of Strathclyde, 295 Cathedral Street, Glasgow, G1 1XL, Scotland, United Kingdom; University of Monash, Australia

**Keywords:** pharmaceutical, salt selection, protonation, crystal structure, pro­ton­ated thio­amide, carbocation

## Abstract

Treating the thio­amide 1-(2,6-di­methyl­phen­yl)thio­urea (C_9_H_12_N_2_S, **DMPT**) with concentrated aqueous strong acids gave three salt forms with protonation at the thio­amide S atom. The structures of 1-(2,6-di­methyl­phen­yl)thio­uro­nium chloride, C_9_H_13_N_2_S^+^·Cl^−^ or **[DMPT(H)][Cl]**, 1-(2,6-di­methyl­phen­yl)thio­uro­nium bromide, C_9_H_13_N_2_S^+^·Br^−^ or **[DMPT(H)][Br]**, and 1-(2,6-di­methyl­phen­yl)thio­uro­nium hy­dro­gen sulfate, C_9_H_13_N_2_S^+^·HSO_4_^−^ or **[DMPT(H)][HSO_4_]**, are com­pared with that of neutral **DMPT**. Protonation at the S atom results in a systematic lengthening of the C=S bond and a shortening of the C—N bonds, with a larger effect found for the bonds involving the secondary amine rather than the primary amine function. The **[DMPT(H)]^+^** cations are found to adopt one of two possible con­for­mations, with the halide species having secondary N—H *syn* to S—H, whilst in the hy­dro­gen sulfate structure, this relationship is *anti*. The change in cation con­for­mation is related to a change in the hy­dro­gen-bonding motif, but all three salt forms form layered structures with two-dimensional hy­dro­gen-bonded units separated by layers of hydro­phobic arene rings. An alternative product type was isolated on adding **DMPT** to concentrated sulfuric or tetra­fluoro­boric acid. Structures of the dicarbocationic di­sulfide species (disulfane-1,2-di­yl)bis­[*N*-(2,6-di­methyl­phen­yl)methane­di­am­in­ium] bis­(tetra­fluoro­borate), C_18_H_24_N_4_S_2_^2+^·2BF_4_^−^ or **[Dimer][BF_4_]_2_**, and (disulfane-1,2-di­yl)bis­[*N*-(2,6-di­methyl­phen­yl)methane­di­am­in­ium] bis­(hy­dro­gen sulfate) monohydrate, C_18_H_24_N_4_S_2_^2+^·2HSO_4_^−^·H_2_O or **[Dimer][HSO_4_]_2_·H_2_O**, are also described.

## Abstract 2

Treating the thio­amide 1-(2,6-di­methyl­phen­yl)thio­urea (**DMPT**) with concentrated aqueous strong acids gave three salt forms with protonation at the thio­amide S atom. Protonation causes lengthening of the C=S bond and shortening of the C—N bonds in effects similar to those seen when amides are pro­ton­ated at the O atom.

## Introduction

**Figure 1:**
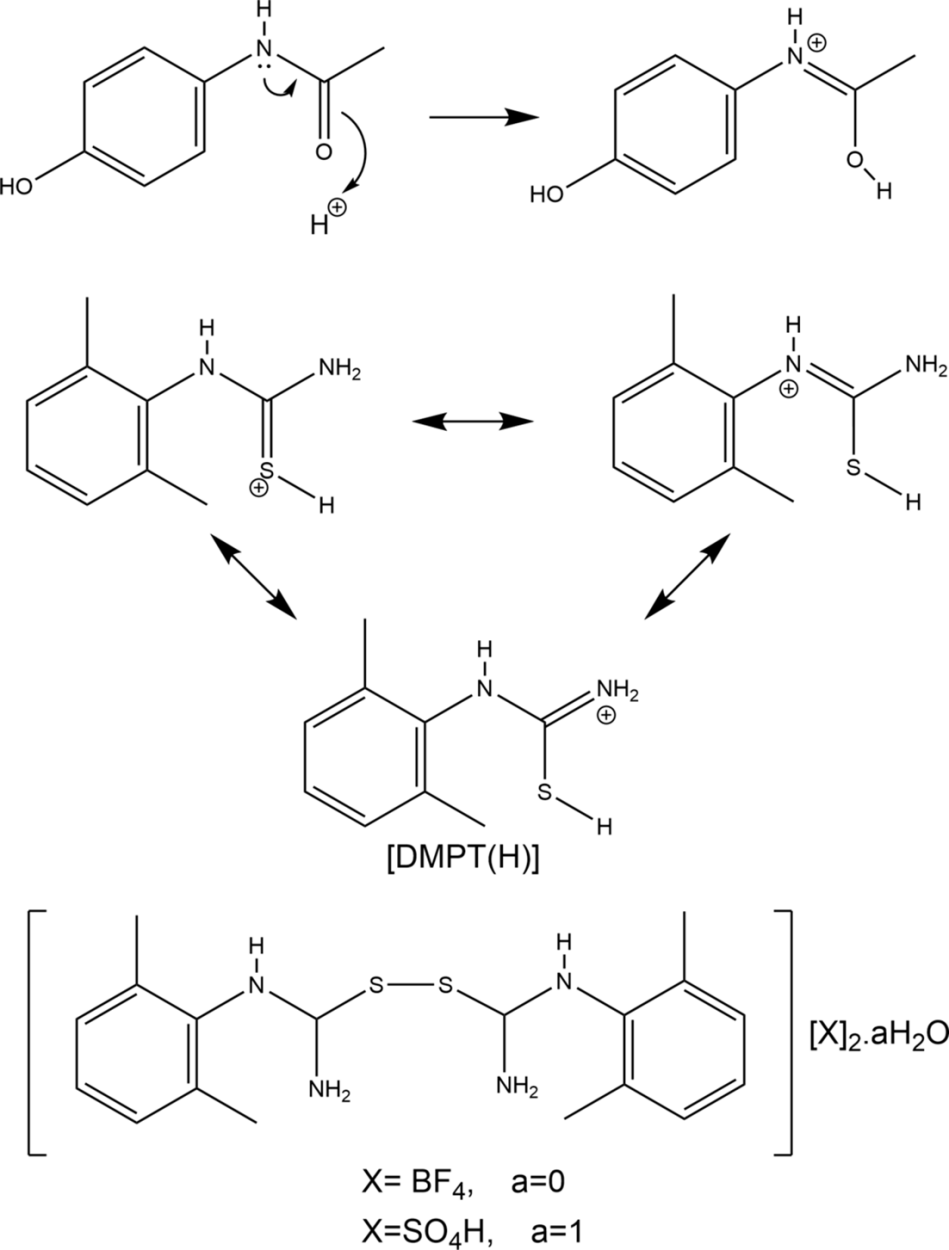
(Top) C=O bond lengthening and C—N bond shortening on protonation of the simple API paracetamol. (Middle) Potential resonance forms of the pro­ton­ated cation **[DMPT(H)]^+^**. (Bottom) The structures of **[Dimer][BF_4_]_2_** and **[Dimer][HSO_4_]_2_·H_2_O**.

Study of pro­ton­ated small mol­ecules from chemical classes often thought of as non-basic (or neutral or non-ionizable) is often undertaken to aid understanding of acid-catalysed reaction mechanisms. Crystallographic studies of such reactive species include those on pro­ton­ated ketones (Uran & Lozinšek, 2025 ▸; Stuart *et al.*, 2017 ▸), pro­ton­ated aldehydes (Stuart *et al.*, 2017 ▸; Hayatifar *et al.*, 2014 ▸), pro­ton­ated esters (Hollenwäger *et al.*, 2025 ▸), pro­ton­ated carb­oxy­lic acids (Hollenwäger *et al.*, 2024 ▸) and pro­ton­ated nitriles (Haiges *et al.*, 2016 ▸). When it comes to the protonation of amides, similar studies have also been carried out and are often performed with a view to gaining an understanding of salt formation for the pharmaceutical industry. Salt forms of active pharmaceutical ingredients (APIs) are commonly generated in an attempt to access a phase of an acidic or basic API which has idealized material properties (Stahl & Wermuth, 2008 ▸). More rarely, similar studies are carried out on formally neutral APIs. Nanubolu and co-workers surveyed known crystal structures of pro­ton­ated amides in 2012 ▸. Since then, systematic API-relevant work on the solid-state structures of pro­ton­ated amides has concentrated on simple amides such as paracetamol cognates (Perumalla & Sun, 2012 ▸; Perumalla *et al.*, 2012 ▸; Trzybiński *et al.*, 2016 ▸; Kennedy *et al.*, 2018 ▸; Suzuki *et al.*, 2020 ▸; Jaconelli & Kennedy, 2024 ▸) and on the urea-containing carbamazepine and its relatives (Perumalla & Sun, 2012 ▸; Perumalla & Sun, 2013 ▸; Buist *et al.*, 2013 ▸; Buist *et al.*, 2015 ▸; Eberlin *et al.*, 2013 ▸; Buist & Kennedy, 2016 ▸). These amides have been shown to protonate at the O atom and to feature patterns of elongated C=O and shortened C—N bonds consistent with the resonance forms shown in Fig. 1 ▸. However, little similar structural work has been undertaken for com­parable thio­amide com­pounds. A search of the Cambridge Structural Database (Version 6.01, with updates to November 2025; Groom *et al.*, 2016 ▸) found 18 structures that contained pro­ton­ated thio­urea [HSC(NH_2_)_2_], but in all structures this cation was associated with small inorganic anions or *MX_x_* anions (*M* = transition metal and *X* = halide) and so relevance to APIs is low (for typical examples, see Biesiada *et al.*, 2014 ▸; Daub & Hillebrecht, 2021 ▸; Li & Li, 2014 ▸). Structures of larger organic fragments that could reliably be described as pro­ton­ated thio­amide anions are restricted to CSD refcodes MUSDAI, PAJLIY, VOZGUP, WEXGUB, XEYVUW and XEYVOQ (Ho *et al.*, 2020 ▸; Liu *et al.*, 2017 ▸; Eichele *et al.*, 2019 ▸; Gladii *et al.*, 1994 ▸; Golovnev *et al.*, 2023 ▸).

To obtain a series of salt forms of pro­ton­ated thio­amides, we investigated protonation of 1-(2,6-di­methyl­phen­yl)thio­urea (**DMPT**). This com­pound was of inter­est partly as it is a simple model com­pound containing an aromatic thio­urea functionality and partly as it is a crucial inter­mediate in the synthesis of the veterinary tranquilizer and drug of abuse xylazine (Ruiz-Colón *et al.*, 2014 ▸; Chang *et al.*, 2025 ▸). The structure of **DMPT** was reported by Sarojini and co-workers in 2007 ▸. Herein we re-elucidate this structure and present the new crystal structures of three pro­ton­ated or salt forms of **DMPT**, namely, **[DMPT(H)][Cl]**, **[DMPT(H)][Br]** and **[DMPT(H)][HSO_4_]**. Also described are the structures of two di­sulfide com­pounds recovered from acidic solutions of **DMPT**; these are **[Dimer][BF_4_]_2_** and **[Dimer][HSO_4_]_2_·H_2_O**.

## Experimental

### Synthesis and crystallization

The synthesis of **DMPT** was based on the method of Burke & Fitzgerald (1989 ▸). Acetone (25 ml) was dried over calcium chloride and ammonium thio­cyanate was dried in a vacuum oven at 80 °C. Ammonium thio­cyanate (5.30 g, 0.07 mol) was added to a two-necked flask fitted with a condenser and dropping funnel, and then dissolved in the dry acetone. Benzoyl chloride (6.7 ml, 0.06 mol) was added at a fast rate, *via* the dropping funnel, to the stirred reaction mixture. Once the addition was com­plete, the dropping funnel was used to add 2,6-di­methyl­aniline (6.2 ml, 0.05 mol) at a rate which caused the reaction mixture to reflux gently. After addition, the mixture was refluxed for a further 5 min. Cooling the reaction mixture to 10 °C gave a solid that was recovered by filtration and washed with water. The crude solid was added to an aqueous solution of 2.8 *M* sodium hydroxide (7.5 g in 67.5 ml), heated to boiling and left for 5 min. After allowing the mixture to cool to room tem­per­a­ture, the resulting solid was filtered off and washed with water. Drying the product under vacuum gave a free-flowing pale-yellow powder (typical yield 6.04 g, 66.5%). Large crystals of **DMPT** were obtained upon recrystallization from hot acetone (m.p. 179–180 °C).

Crystals of **[DMPT(H)][Cl]** and **[DMPT(H)][Br]** were produced by first forming slurries from 0.1 g (0.55 mmol) of **DMPT** and 3 ml of deionized water. 2 ml of the appropriate concentrated acid were then added slowly and the solutions warmed to aid dissolution. After filtration, the clear solutions obtained were left to evaporate slowly, giving suitable colourless crystals within 4 d. The melting points were 167–168 and 145–146 °C for the chloride and bromide salt, respectively.

Using the same method with concentrated sulfuric acid initially gave crystals of the expected product **[DMPT(H)][HSO_4_]** after approximately six weeks of slow evaporation. However, on rechecking the same acidic–aqueous sample after approximately six months, the crystals isolated were found to be **[Dimer][HSO_4_]_2_·H_2_O**. Similar checks on the halide salt samples (above) observed no such di­sulfide products, with crystals of both **[DMPT(H)][Cl]** and **[DMPT(H)][Br]** still being present despite being in contact with the original acidic–aqueous mother liquors.

Using a similar method with concentrated HBF_4_ did not give any of the desired **[DMPT(H)]^+^**-containing product. Instead, only large colourless crystals of the di­sulfide product **[Dimer][BF_4_]_2_** were isolated. These became apparent after approximately 7 d of slow evaporation.

### Refinement

Crystal data, data collection and structure refinement details are summarized in Table 1 ▸. H atoms bonded to C atoms were observed in difference electron-density calculations, but were added in expected positions and refined as riding on their parent C atoms. All H atoms bonded to S atoms were refined freely and isotropically. Similar free refinement was used for most H atoms bonded to either O or N atoms. However, N—H distances in both **[DMPT(H)][Cl]** and **[DMPT(H)][HSO_4_]** were restrained to 0.88 (1) Å, as were both the N—H and the O—H distances in **[Dimer][HSO_4_]_2_·H_2_O**. The H atoms of the methyl group at C8 of **DMPT** were modelled as rotationally disordered over two sites.

## Results and discussion

**Figure 2:**
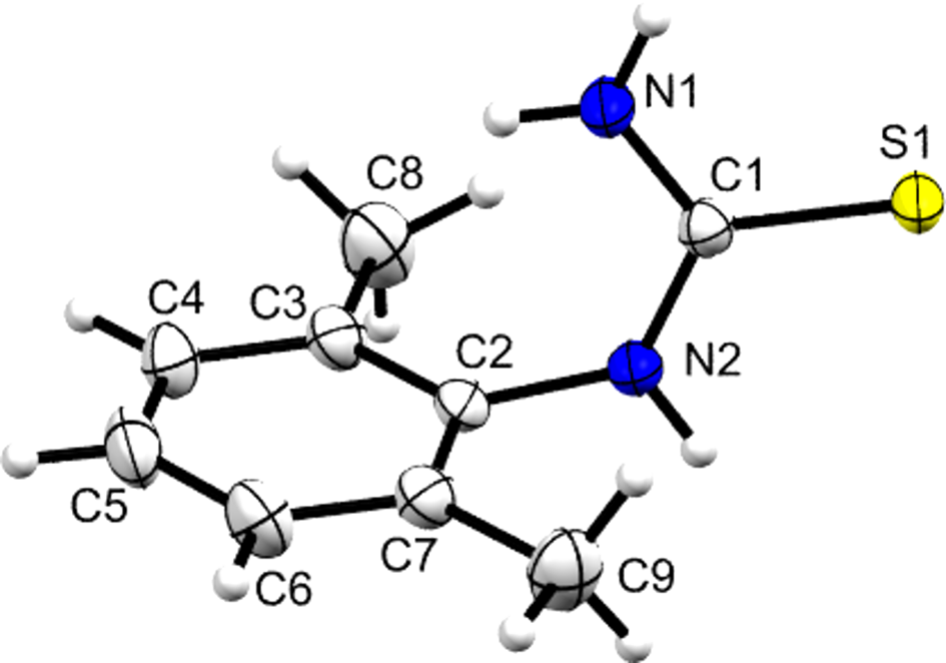
The mol­ecular structure of **DMPT**, with displacement ellipsoids drawn at the 50% probability level. H atoms are drawn as small spheres of arbitrary size. Disordered H-atom positions on C8 have been omitted for clarity.

**Figure 3:**
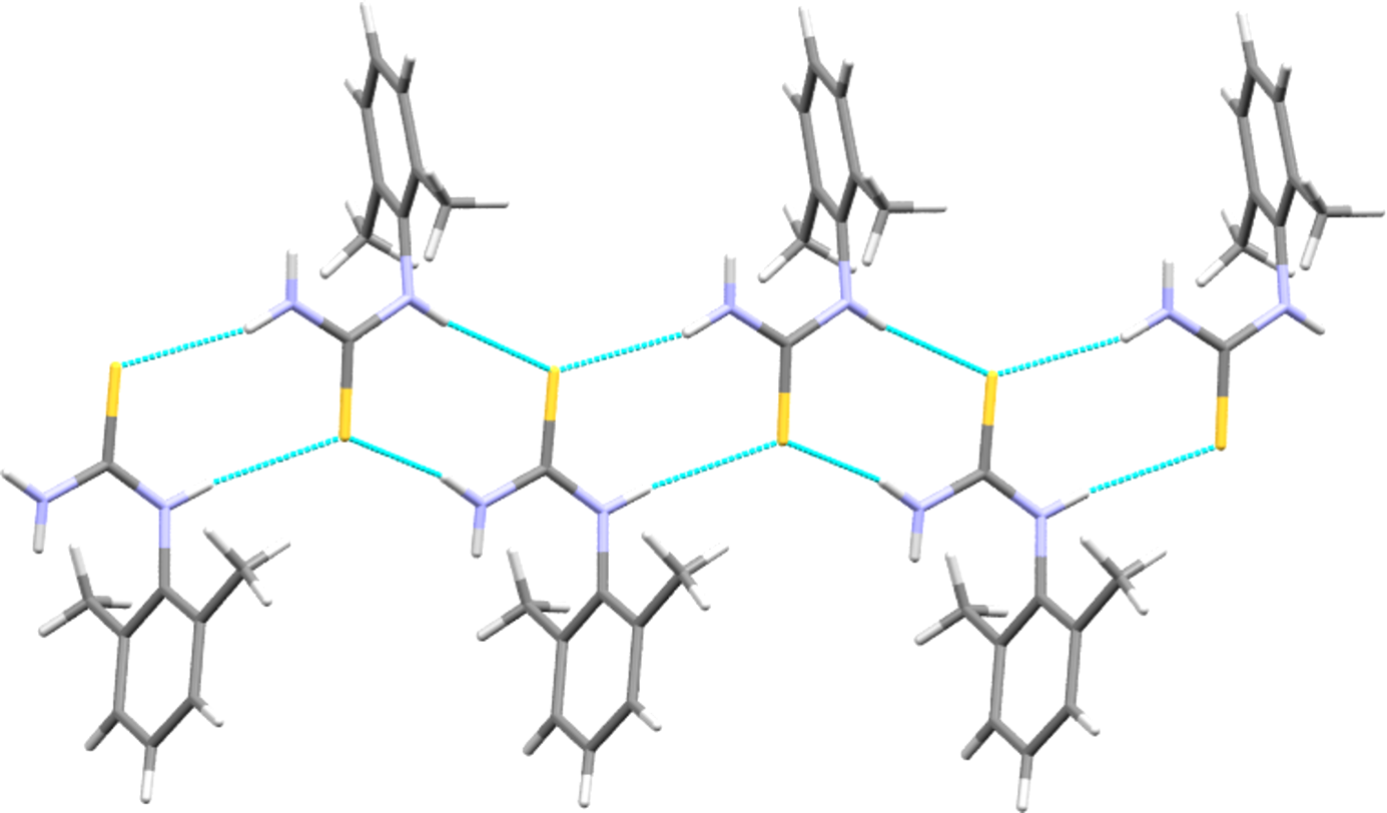
Section of the hy­dro­gen-bonded (dashed lines) one-dimensional ribbon found in the structure of **DMPT**.

The mol­ecular structure of **DMPT** is shown in Fig. 2 ▸ and is in agreement with that reported earlier by Sarojini *et al.* (2007 ▸). This structure is used herein largely as a com­parison for the species with pro­ton­ated **[DMPT(H)]^+^** cations, but some salient points to note are that there is an 80.82 (5)° angle between the plane of the thio­amide group and the aromatic ring plane (here and later defined by the N1/C1/S1/N2 plane and the six C atoms C2–C7, respectively) and that both the N atoms adopt essentially *sp*^2^ geometries coplanar with the thio­amide plane, although there is a small nonplanar deviation of the secondary amine (N2), with atom C2 lying 0.3246 (17) Å from the thio­amide plane. The main inter­molecular feature is the one-dimensional hy­dro­gen-bonded ribbon shown in Fig. 3 ▸. This extends parallel to the crystallographic *b* direction and is formed from 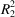
(8) motifs utilizing sulfur as the acceptor and two of the three N—H atoms as donors; see Table 2 ▸ for further details (Etter *et al.*, 1990 ▸). Notably, this arrangement does not allow the third N—H atom, on the primary amine, to act as a hy­dro­gen-bond donor.

**Figure 4:**
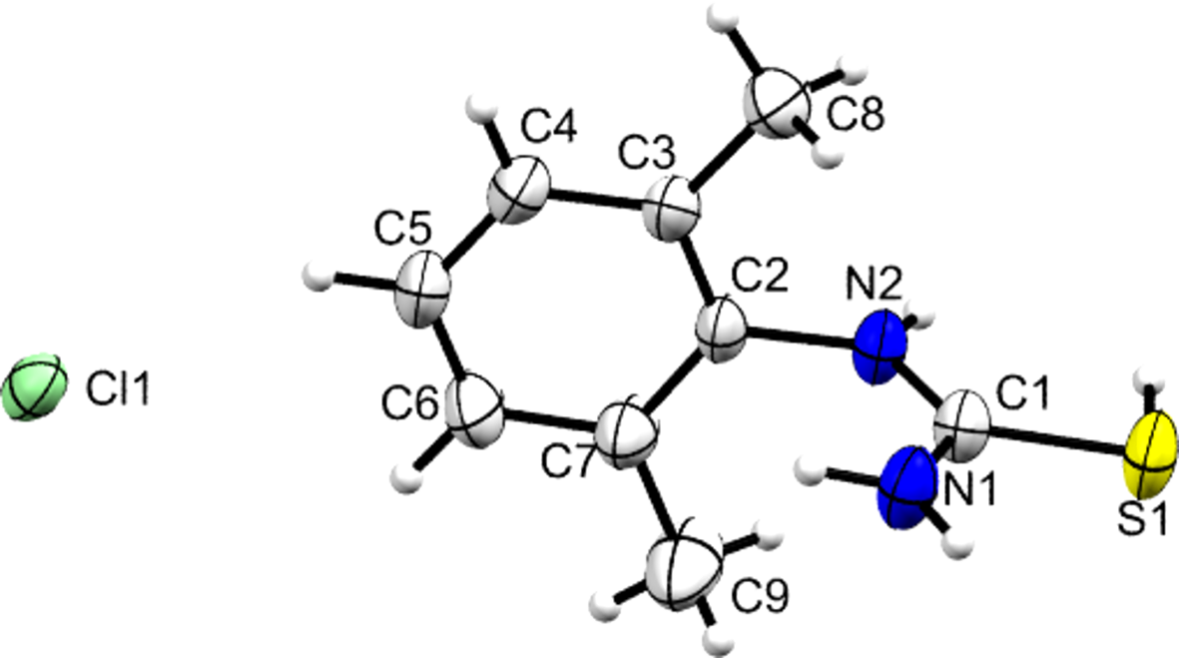
Contents of the asymmetric unit of **[DMPT(H)][Cl]**, with displacement ellipsoids drawn at the 50% probability level. H atoms are drawn as small spheres of arbitrary size.

Addition of concentrated HCl to a slurry of **DMPT** gave colourless crystals identified as **[DMPT(H)][Cl]**. It was noted that at tem­per­a­tures below 170 K, these crystals did not diffract – and that often the mounted single crystal shattered. This is suggestive of the chloride salt displaying a tem­per­a­ture-induced phase change. Reactions with HBr and H_2_SO_4_ also gave crystals that contained the **[DMPT(H)]^+^** cation and the structures of all three species **[DMPT(H)][Cl]**, **[DMPT(H)][Br]** and **[DMPT(H)][HSO_4_]** are displayed in Figs. 4 ▸–6 ▸ ▸.

In all cases, the protonation site of **DMPT** is identified as the S atom. Electron-density contour maps in support of these protonation sites are available in the supporting information. For the halide salts, the pro­ton­ated S—H group is *syn* to the N—H group of the secondary amide at N2 and neutral **DMPT** has a similar *syn* con­for­mation. Conversely, in the hy­dro­gen sulfate salt, the S—H group is orientated *anti* to the N2 N—H moiety. In terms of the dihedral angles between the thio­amide plane and the aromatic plane, there is no systematic change observed between the **[DMPT(H)]^+^** cations and **DMPT**, with the value of 80.81 (5)° for the latter falling within the range [75.10 (4)–89.94 (5)°] found for the cations. A small difference is that the slight pyramidalization of the secondary amine seen for **DMPT** is not present in any of the cationic forms. In all cases, the C1—S1—H1*S* angle of the cation is somewhat narrow [range 92 (2)–95.9 (11)°]. However, this is supported by the inter­nal consistency of the three reported **[DMPT(H)]^+^** structures and by similar angles being observed in other pro­ton­ated thio­amides (Ho *et al.*, 2020 ▸; Liu *et al.*, 2017 ▸; Golovnev *et al.*, 2023 ▸). From consideration of resonance structures (Fig. 1 ▸) and by com­parison to known O-pro­ton­ated amide structures, it is expected that the C1=S1 bond lengths will increase upon protonation at S and that the N—C1 bonds should correspondingly shorten. Table 3 ▸ shows that this effect is indeed observed here, with protonation leading to the C1=S1 distances increasing by 0.052 (2) to 0.054 (2) Å, C1—N1 decreasing by 0.007 (4) to 0.015 (3) Å and C1—N2 decreasing by 0.026 (3) to 0.032 (3) Å. These values indicate that the increase in double-bond character is much larger for the C1—N2 bond of the secondary amine moiety bonded to the aryl ring, than it is for the C1—N1 bond to the primary amine. Similar effects were seen in the pro­ton­ated urea functionality of carbamazepine derivatives [*R*_2_NC(OH)NH_2_], where bond-length shortening was more pronounced for the N—C bond of the tertiary amine than for the primary amine (Buist *et al.*, 2013 ▸). At approximately 0.05 Å, the increase in the C=S bond lengths is greater in absolute terms than that seen for hemi-pro­ton­ated amides (*e.g.* for acetanilide, **ACT**, the C=O bond lengths increase by approximately 0.02 to 0.04 Å on forming **[ACT(H)·ACT]** pairs), but at the low end for the typical increase seen for fully pro­ton­ated amide species (*e.g.* for carbamazepine and paracetamol, C=O bond lengths typically increase by 0.05 to 0.07 Å and, in all cases, these changes are at least an order of magnitude greater than the associated error) (Jaconelli & Kennedy, 2024 ▸; Buist *et al.*, 2013 ▸; Kennedy *et al.*, 2018 ▸). This is inter­esting as C=S bonds are of course longer than C=O bonds and thus the observed increase in bond length between neutral and pro­ton­ated states is smaller in percentage terms (approximately 3% com­pared to 5%) for C=S *versus* C=O. It is suggested that the relatively small effect may be related to the poorer 2*p*–3*p* orbital overlap between C and S in forming the π-bond, as com­pared to the more energy matched 2*p*–2*p* orbital overlap in the O and C π-bond. Overall this renders the ‘double-bond’ description of C=S somewhat less relevant that it is for C=O. The C=S bond lengths observed here fit with those found for com­pounds containing the thio­urea hy­dro­gen ion (HSCNH_2_)_2_, albeit at the long end of the observed range (*e.g.* Biesiada *et al.*, 2014 ▸; Daub & Hillebrecht, 2021 ▸; Li & Li, 2014 ▸).

Table 4 ▸ illustrates that protonation also results in a systematic change to the bond angles of the thio­amide. The C1—N2—C2 and N1—C1—N2 angles both increase on protonation, by approximately 2.0–2.5 and 3.7–4.1°, respectively, with all error values an order of magnitude lower. The change in the angle at C1 is matched by similar increases in the equivalent amide species (Jaconelli & Kennedy, 2024 ▸; Buist *et al.*, 2013 ▸). However, the other angles present more com­plex behaviour. For both paracetamol and acetanilide, the equivalent of the C1—N2—C2 angle was found to change in different ways depending on the con­for­mation of the cation (Jaconelli & Kennedy, 2024 ▸; Kennedy *et al.*, 2018 ▸). When the amide was coplanar with the aryl ring, this angle systematically widened on protonation, but narrowed when the amide group was twisted out of the plane of the ring. All the current pro­ton­ated thio­amide fragments are twisted (see Table 4 ▸), but in contrast to the amide examples all show widening of C1—N2—C2. The angular changes that seem to depend on con­for­mation here are those involving the S atom. For the two halide species where the pro­ton­ated S—H group is *syn* to the N—H group of the secondary amide, N1—C1—S1 decreases on protonation [from 120.35 (10) to 117.69 (12) and 117.21 (12)°], as do the N2—C1—S1 angles, albeit by a smaller amount [from 121.66 (10) to 120.29 (12) and 121.08 (12)°]. In contrast in the *anti* species, **[DMPT(H)][HSO_4_]**, N1—C1—S1 increases to 121.1 (2)° and N2—C1—S1 shows a large decrease to 116.8 (2)°. Thus, angular changes on protonation of this thio­amide are found to depend upon con­for­mation, as well as on the act of protonation itself.

**Figure 7:**
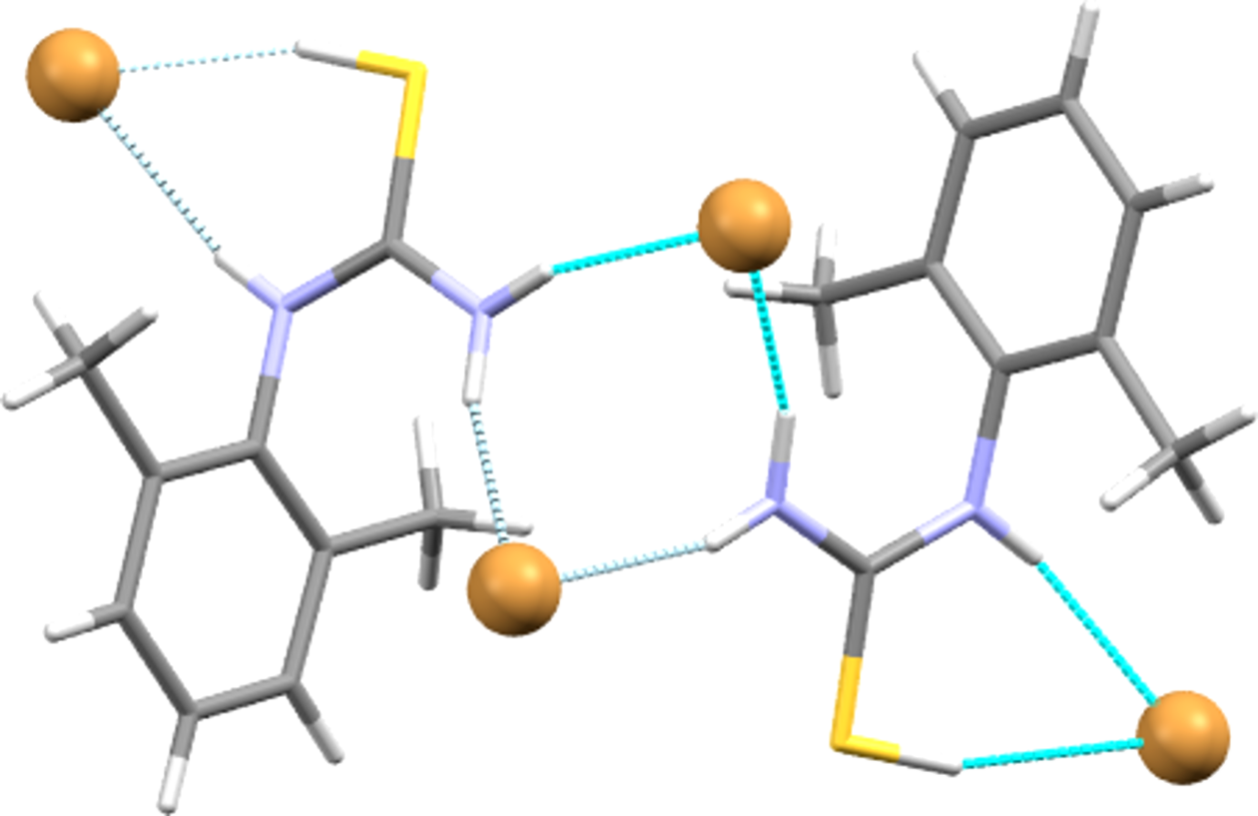
Section illustrating the main hy­dro­gen-bonding inter­action types (dashed lines) found in **[DMPT(H)][Br]**. The same structural motifs are found in **[DMPT(H)][Cl]**. Cations are drawn in stick format and bromide anions are shown as large balls.

**Figure 8:**
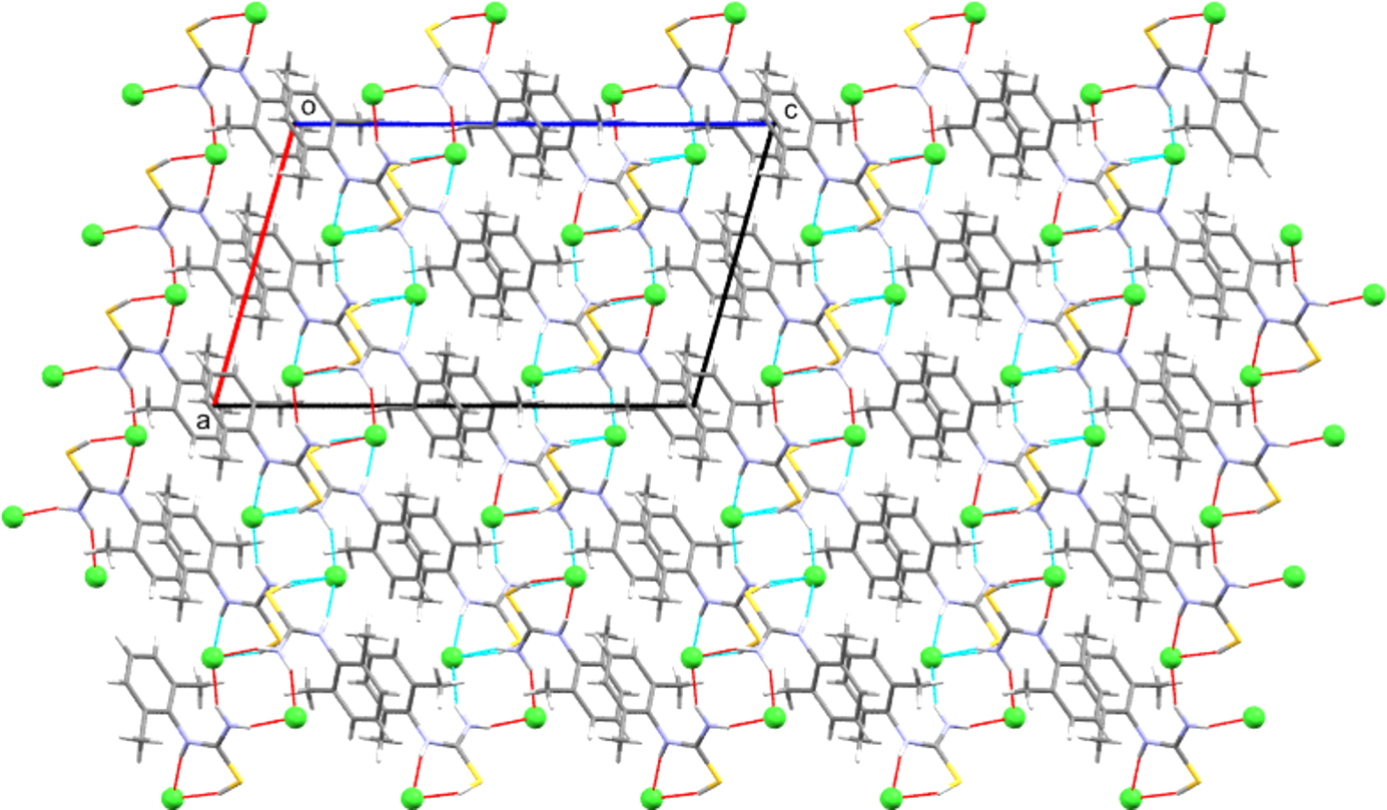
Packing array found in **[DMPT(H)][Cl]**, viewed along the *b* direction. Note the hydro­phobic layers consisting of aromatic groups that separate the hy­dro­gen-bonded hydro­philic zones. Both layer types lie parallel to the *ab* plane.

The *syn* arrangement of the pro­ton­ated S—H group and the N—H group of the secondary amide allow these two groups to form a six-membered hy­dro­gen-bonded ring, 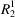
(6), with the halide anions of **[DMPT(H)][Cl]** and **[DMPT(H)][Br]**. In both structures, the two H atoms of the NH_2_ groups also act as single hy­dro­gen-bond donors to two further halide anions. The action of the crystallographic twofold axis forms [NH_2_–*X*–NH_2_–*X*] dimers of type 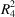
(8) from these latter inter­actions (see Table 2 ▸ and Fig. 7 ▸). Combining these inter­action types gives two-dimensional hy­dro­gen-bonded layers that lie per­pen­dic­u­lar to the *c* axis. Each such two-dimensional motif is separated from its neighbours by the aromatic groups, giving layered structures, such as that seen for **[DMPT(H)][Cl]** in Fig. 8 ▸.

**Figure 9:**
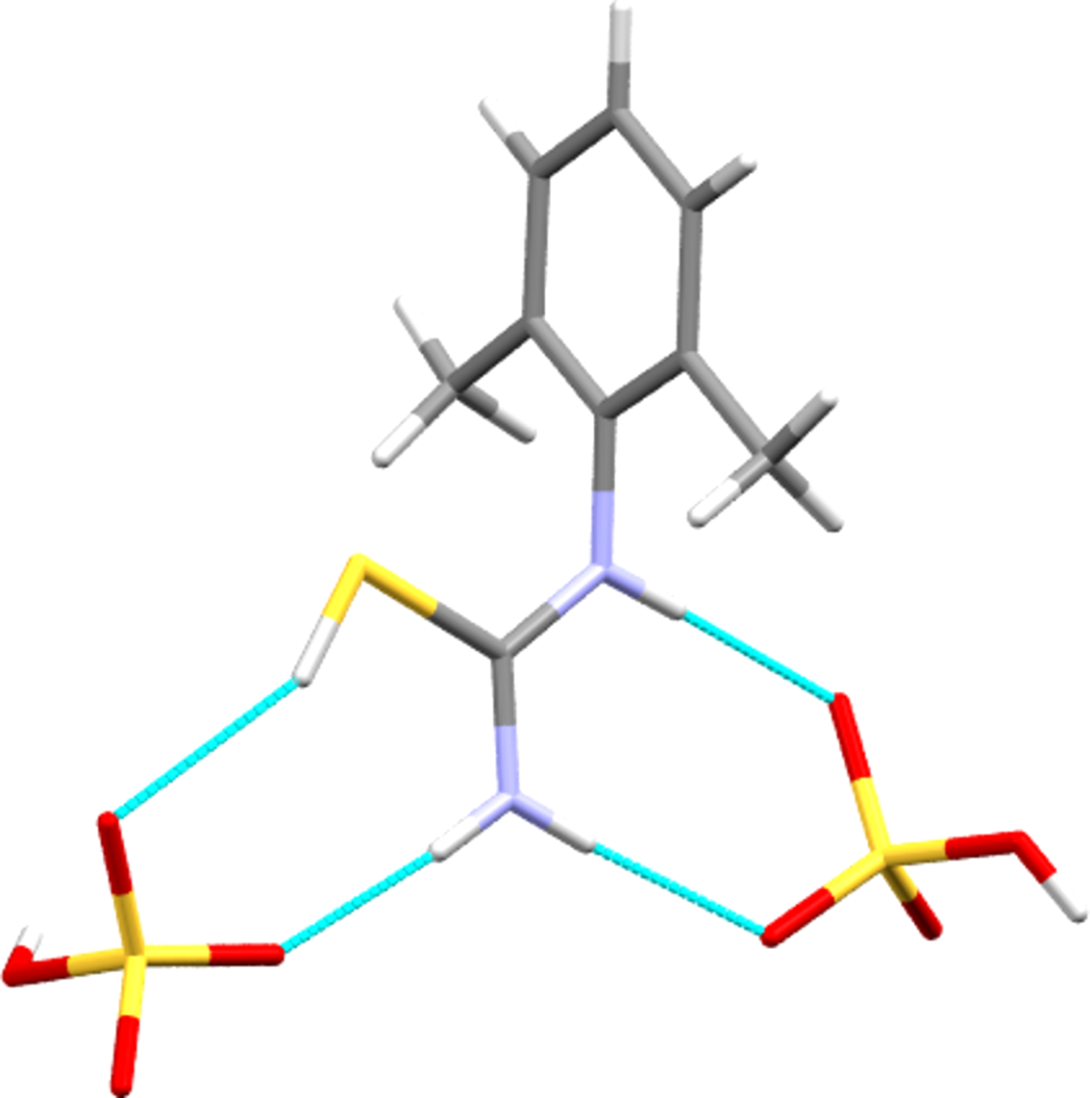
Hydrogen-bonded contacts (dashed lines) formed by the cation in **[DMPT(H)][HSO_4_]**.

**Figure 10:**
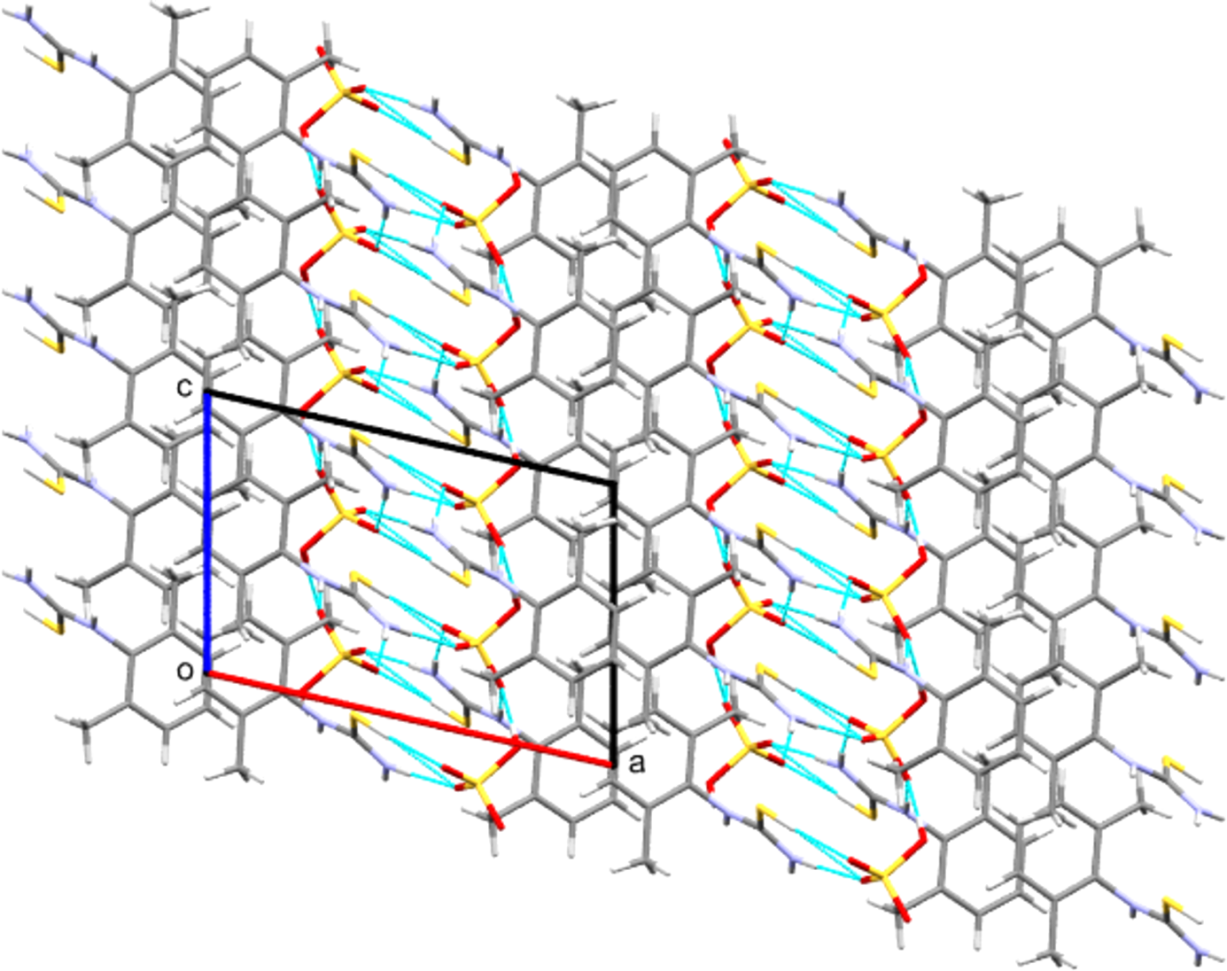
Packing diagram of the **[DMPT(H)][HSO_4_]** structure, viewed along the direction of the *b* axis.

The *anti* conformer of the cation in **[DMPT(H)][HSO_4_]** forms one hy­dro­gen bond from each donor atom to four O atoms of two neighbouring hy­dro­gen sulfate anions. This gives two 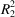
(8) rings, one with S—H and N—H donors, and the other with two N—H donors, as shown in Fig. 9 ▸ and detailed in Table 2 ▸. These inter­actions combine with anion-to-anion O—H⋯O hy­dro­gen bonds to give a two-dimensional hy­dro­gen-bonded construct that forms layers perpendicular to the crystallographic *a* direction (see Fig. 10 ▸). As with the halide structures above, these layers are separated by layers formed by the hydro­phobic aromatic groups.

**Figure 11:**
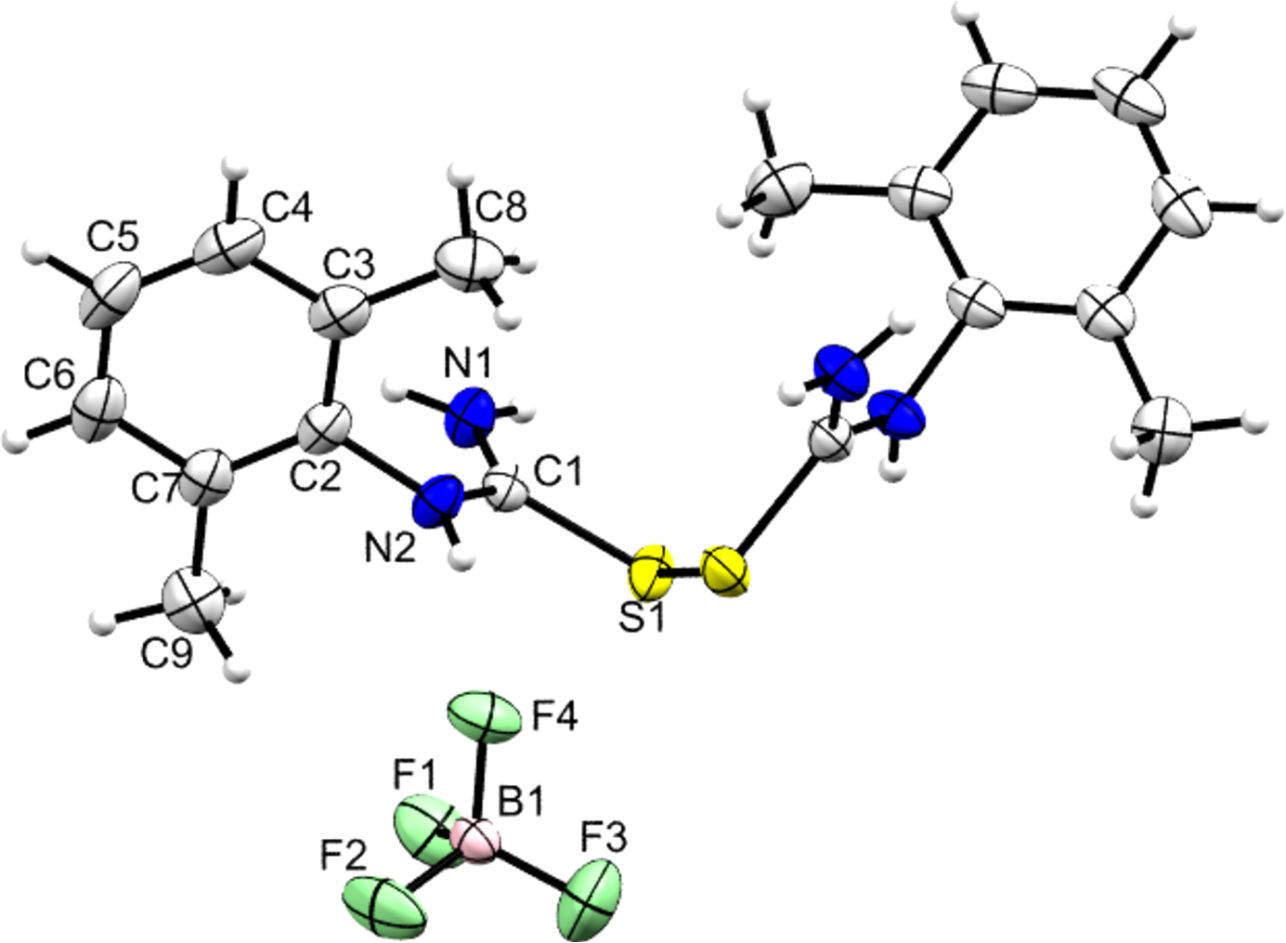
The asymmetric unit of **[Dimer][BF_4_]_2_**, with the cation expanded to show its full atomic connectivity. The unnumbered atoms are related to the named atoms by the operation (−*x* + 1, *y*, −*z* + 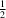). Displacement ellipsoids are drawn at the 50% probability level. H atoms are drawn as small spheres of arbitrary size.

Two further products were isolated from addition of strong acids to **DMPT**, as described in the *Experimental* section. These were the di­sulfide species **[Dimer][BF_4_]_2_** and **[Dimer][HSO_4_]_2_·H_2_O**, whose structures are illustrated in Figs. 11 ▸ and 12 ▸. The asymmetric unit of the BF_4_^−^ salt contains one anion and half of the cation – with the other half generated by the action of a crystallographic twofold axis. Although these are clearly decom­position products and not the expected salt forms of **DMPT**, their structures are reported here as there are surprisingly few structural reports of carbocations with a CSNN core in the CSD. A search found only five structures with acyclic CSNN cores and of these only that of [(Me_2_N)_2_CSSC(NMe_2_)_2_][Fe_2_OCl_6_] contained a dication as described here (Senda *et al.*, 2000 ▸). Note that the synthesis of di­sulfides is of perennial inter­est in medicinally-relevant organic synthesis (*e.g.* Hou *et al.*, 2025 ▸; Hunter *et al.*, 2006 ▸).

The S—S di­sulfide bonds of 2.0364 (8) and 2.0431 (5) Å are indistinguishable from similar bond lengths found in neutral species [a search found 1656 C—S—S—C fragments, in high-quality structures with *R* < 5%, that gave an average S—S bond length of 2.05 (4) Å]. Table 5 ▸ shows that the two salts of the di­sulfide species also have similar C—N and C—S bonds at the carbocation centres, and that both C—S—S—C torsion angles deviate only slightly from a per­pen­dic­u­lar con­for­mation. Further indications of the con­for­mational similarity of the two dicarbocations are that the aromatic ring planes and their adjacent CSN_2_ carbocation planes are essentially perpendicular (range 86.95–87.23°), and that both have broadly similar dihedral angles of 50.42 and 46.32° between the planes of their two aromatic rings.

**Figure 13:**
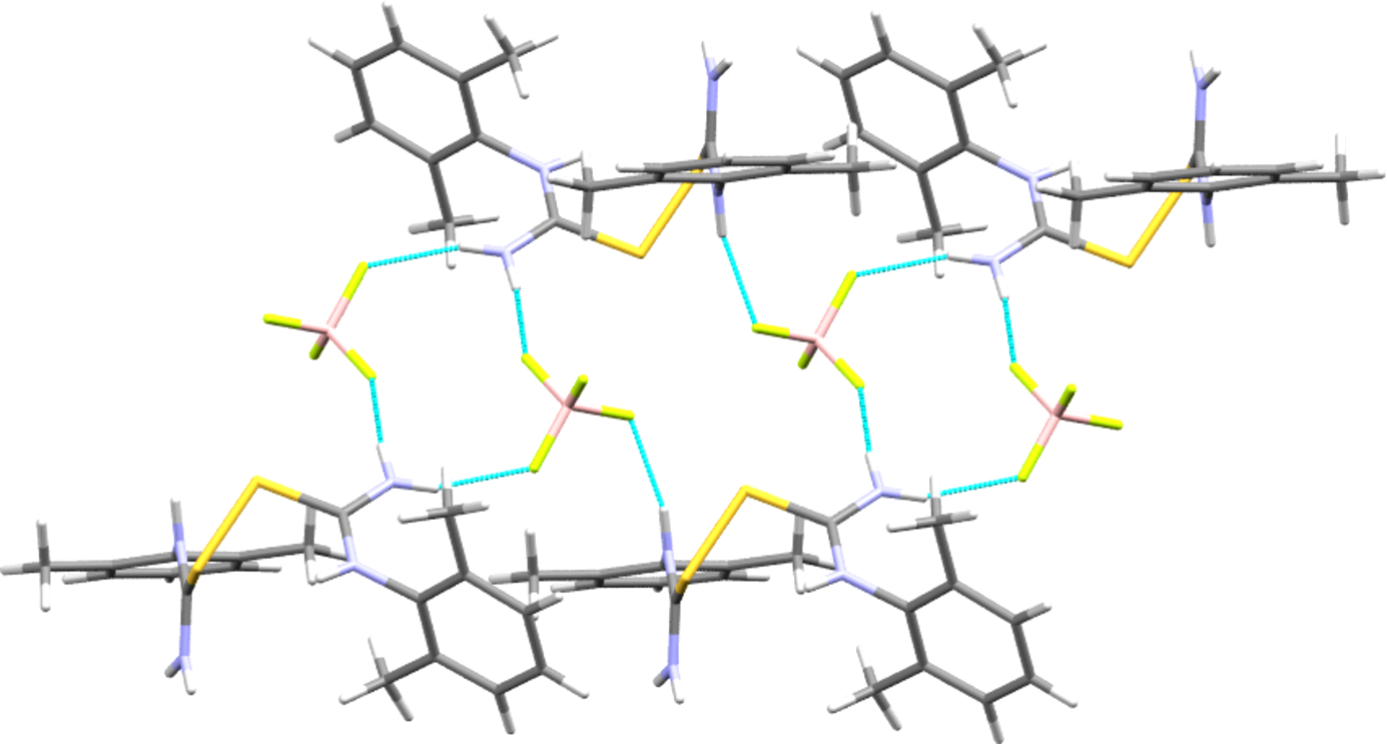
Representation of the hy­dro­gen bonding (dashed lines) found in **[Dimer][BF_4_]_2_**, showing both the 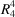
(12) rings common to the BF_4_^−^ and HSO_4_^−^ salt forms, and the 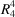
(22) rings found only in the BF_4_^−^ salt.

There are some differences in structure between the two di­sulfide structures related to details of the hy­dro­gen bonding present. Similarities are that both cations form one hy­dro­gen bond from every NH atom to an F or O atom of the appropriate anion, and that in both structures the primary amines form 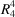
(12) rings involving two NH_2_ groups and two anions. However, the motifs formed by the secondary amine differ. In the BF_4_^−^ salt, the principle motif is 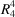
(22) rings formed from two cations and two anions, but the hy­dro­gen-bond donor OH group of the hy­dro­gen sulfate anion ensures a different outcome in **[Dimer][HSO_4_]_2_·H_2_O**. Here, an 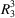
(13) motif is formed from a single cation and two anions. These hy­dro­gen-bonding contacts are illustrated in Figs. 13 ▸ and 14 ▸, and detailed in Table 2 ▸. A final point with respect to inter­molecular contacts is that none of the **[DMPT(H)]^+^**-cation-containing structures show any significant cation-to-cation inter­actions. However, both structures with **[Dimer]^+^** cations do so. Both feature short C⋯C contacts that indicate that all the C_6_ aryl rings are involved in π–π inter­actions [minimum C⋯C distances of 3.343 (3) and 3.224 (4) Å for **[Dimer][BF_4_]_2_** and **[Dimer][HSO_4_]_2_·H_2_O**, respectively].

## Summary

Three salt forms with S-pro­ton­ated thio­amide groups were characterized crystallographically, as were two di­sulfide car­bo­cation species formed from the neutral parent thio­amide. The **[DMPT(H)]^+^** cations were found to have elongated C=S distances and shortened C—N distances com­pared to the structure of neutral **DMPT**. These deviations are in line with changes found previously in O-pro­ton­ated amide species. Systematic changes to the bond angles of the pro­ton­ated thio­amide groups were also observed, but these seemed to be sensitive to the con­for­mation of the group. The species with *syn* con­for­mations gave different narrowing/widening behaviour to that with the *anti* cation con­for­mation. This illustrates that not all structural changes observed on protonation can be simply attributed to the act of protonation itself, subtleties of con­for­mation can alter what may be expected. In all three **[DMPT(H)][*****X*****]** species, hy­dro­gen bonding between the polar thio­amide group and the anions gave two-dimensional hy­dro­gen-bonded constructs. These layers were separated from one another by layers of non-polar di­methyl­phenyl groups.

## Supplementary Material

Crystal structure: contains datablock(s) DMPT, DMPTHCl, DMPTHBr, DMPTHSO4H, DimerBF42, DimerSO4H2.H2O, global. DOI: 10.1107/S2053229626001804/jx3104sup1.cif

Structure factors: contains datablock(s) DMPT. DOI: 10.1107/S2053229626001804/jx3104DMPTsup2.hkl

Structure factors: contains datablock(s) DMPTHCl. DOI: 10.1107/S2053229626001804/jx3104DMPTHClsup3.hkl

Structure factors: contains datablock(s) DMPTHBr. DOI: 10.1107/S2053229626001804/jx3104DMPTHBrsup4.hkl

Structure factors: contains datablock(s) DMPTHSO4H. DOI: 10.1107/S2053229626001804/jx3104DMPTHSO4Hsup5.hkl

Structure factors: contains datablock(s) DimerBF42. DOI: 10.1107/S2053229626001804/jx3104DimerBF42sup6.hkl

Structure factors: contains datablock(s) DimerSO4H2.H2O. DOI: 10.1107/S2053229626001804/jx3104DimerSO4H2.H2Osup7.hkl

Supporting information file. DOI: 10.1107/S2053229626001804/jx3104sup8.pdf

CCDC references: 2531901, 2531900, 2531899, 2531898, 2531897, 2531896

## Figures and Tables

**Figure 5:**
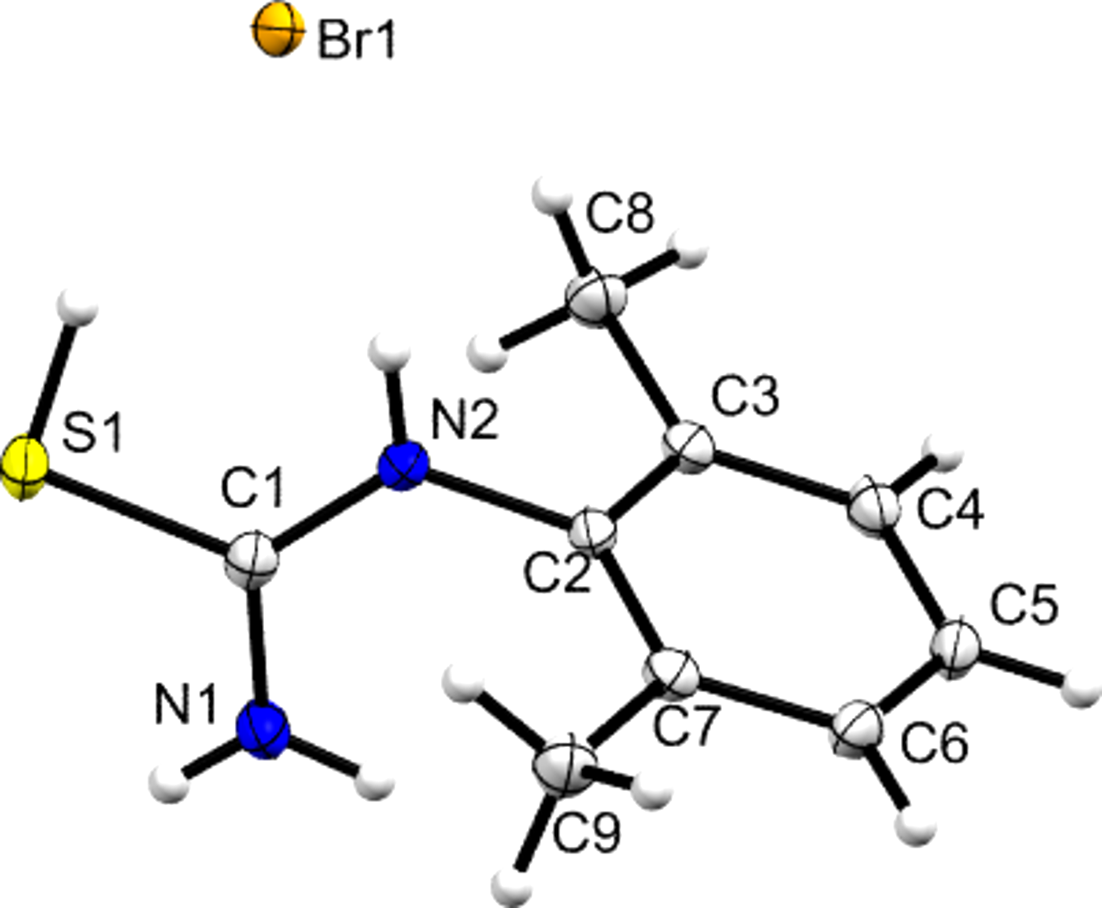
Contents of the asymmetric unit of **[DMPT(H)][Br]**, with displacement ellipsoids drawn at the 50% probability level. H atoms are drawn as small spheres of arbitrary size.

**Figure 6:**
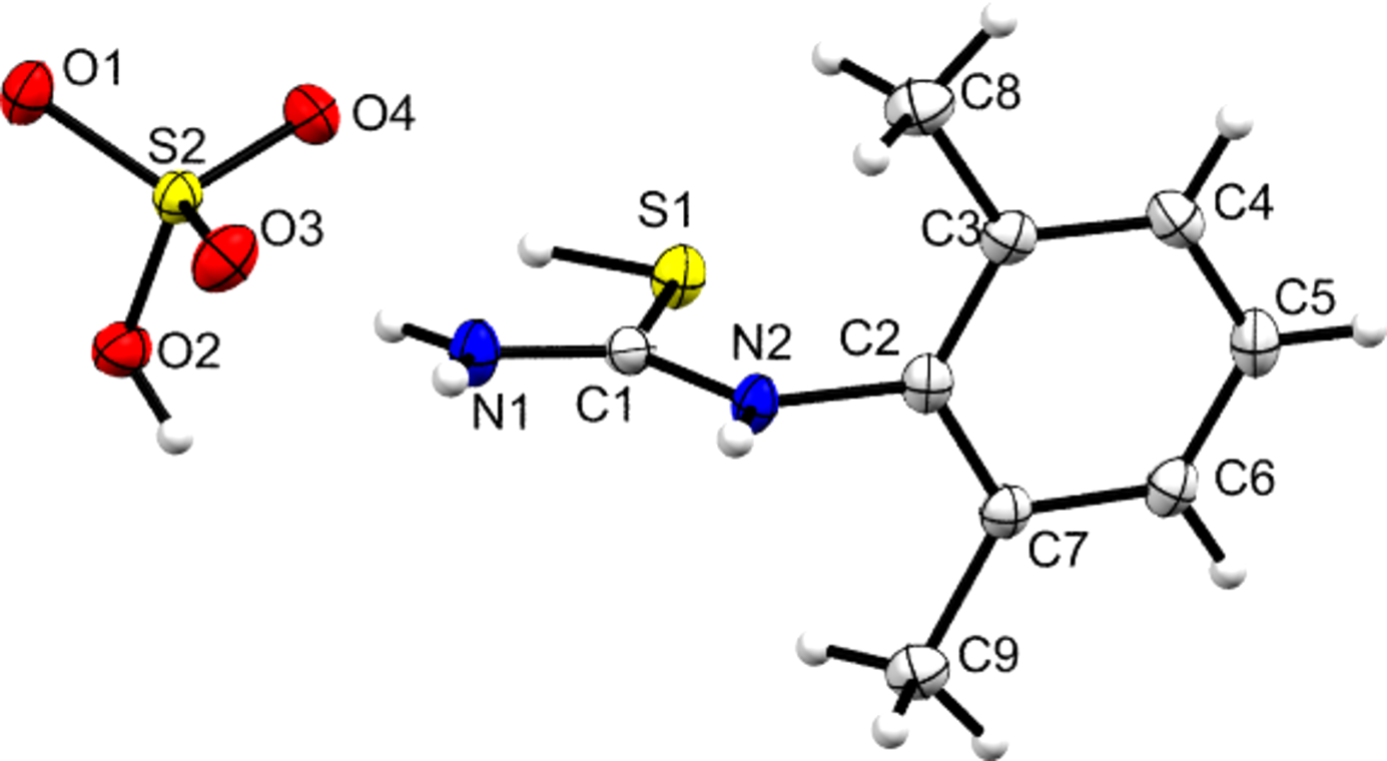
Contents of the asymmetric unit of **[DMPT(H)][HSO_4_]**, with displacement ellipsoids drawn at the 50% probability level. H atoms are drawn as small spheres of arbitrary size.

**Figure 12:**
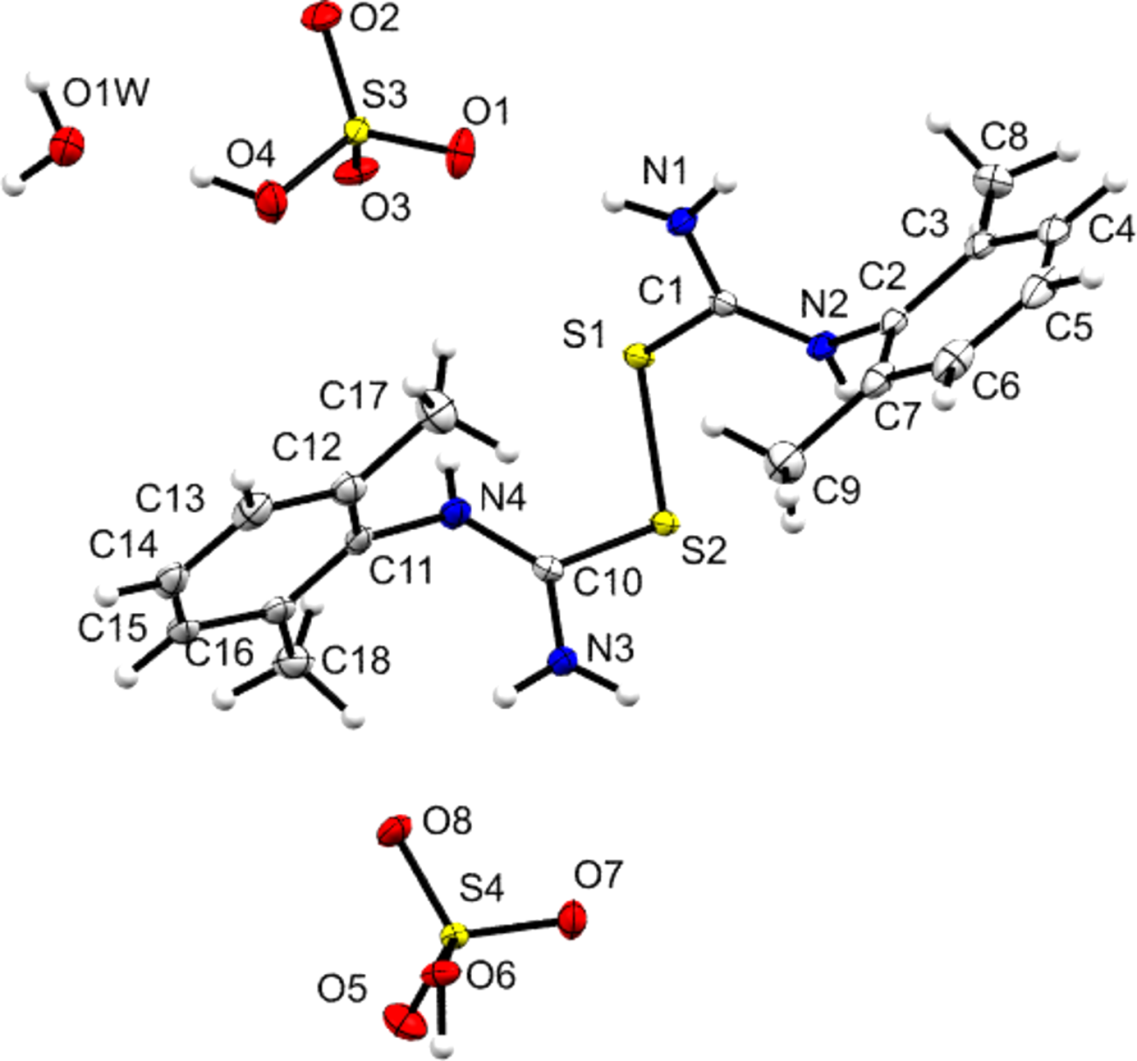
Contents of the asymmetric unit of **[Dimer][HSO_4_]_2_·H_2_O**. Displacement ellipsoids are drawn at the 50% probability level. H atoms are drawn as small spheres of arbitrary size.

**Figure 14:**
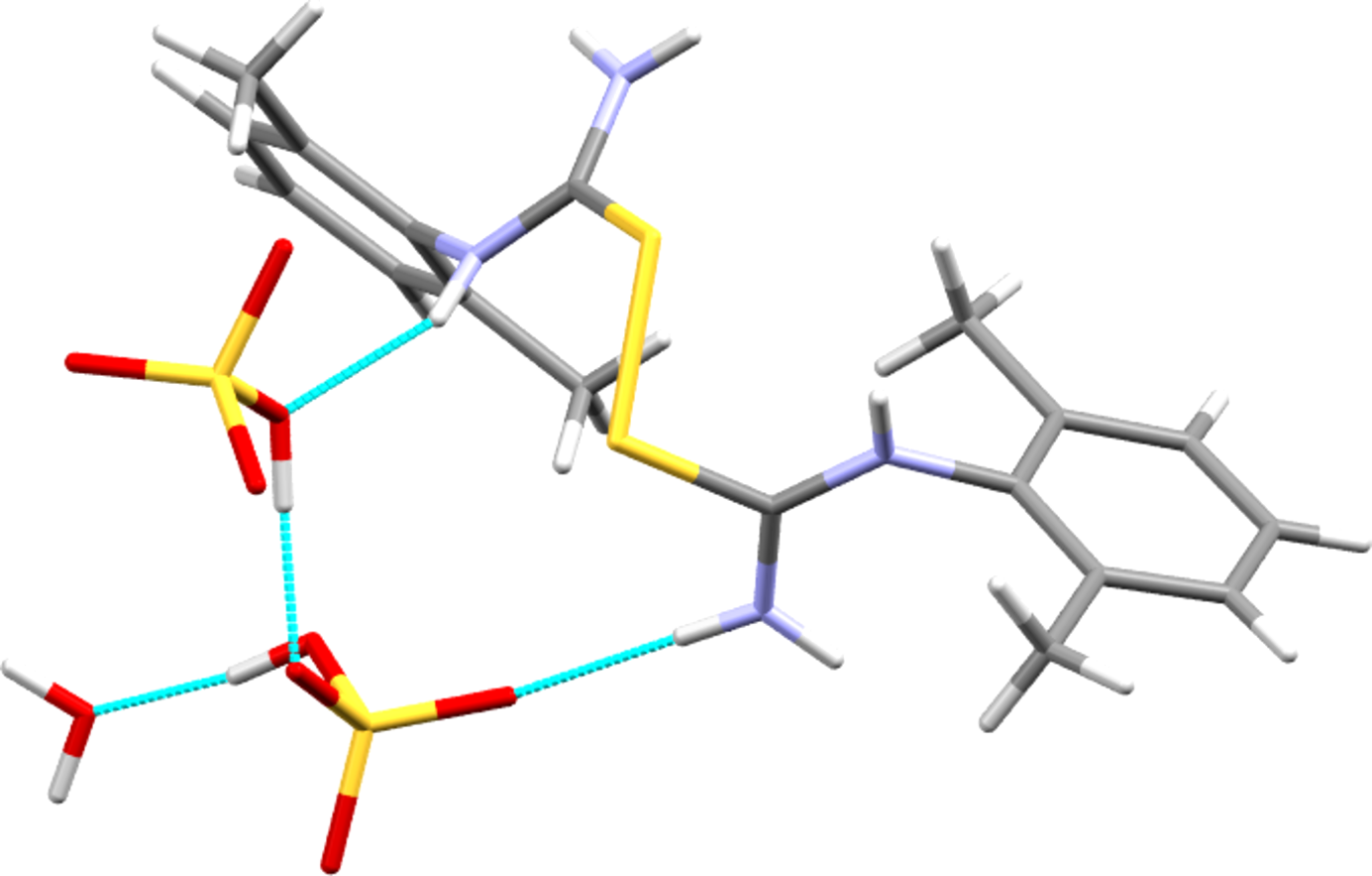
llustration of the 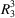
(13) hy­dro­gen-bonding motif found in the structure of **[Dimer][HSO_4_]_2_·H_2_O**. Hydrogen bonds are represented by dashed lines.

**Table:** Experiments were carried out with Cu *K*α radiation using a Rigaku Synergy-i diffractometer. Absorption was corrected for by multi-scan methods (*CrysAlis PRO*; Rigaku OD, 2025 ▸). H atoms were treated by a mixture of independent and constrained refinement.

	**DMPT**	**[DMPT(H)][Cl]**	**[DMPT(H)][Br]**
Crystal data			
Chemical formula	C_9_H_12_N_2_S	C_9_H_13_N_2_S^+^·Cl^−^	C_9_H_13_N_2_S^+^·Br^−^
*M*_r_	180.27	216.72	261.18
Crystal system, space group	Monoclinic, *P*2_1_/*n*	Monoclinic, *C*2/*c*	Monoclinic, *C*2/*c*
Temperature (K)	180	180	100
*a*, *b*, *c* (Å)	9.8193 (1), 8.3655 (3), 11.7827 (2)	11.7848 (2), 10.3644 (2), 19.2687 (3)	13.3544 (2), 8.7556 (1), 20.5020 (2)
β (°)	91.696 (1)	105.844 (2)	112.485 (1)
*V* (Å^3^)	967.45 (4)	2264.11 (7)	2214.97 (5)
*Z*	4	8	8
μ (mm^−1^)	2.54	4.37	6.48
Crystal size (mm)	0.25 × 0.18 × 0.06	0.20 × 0.15 × 0.10	0.17 × 0.14 × 0.08

Data collection			
*T*_min_, *T*_max_	0.758, 1.000	0.758, 1.000	0.770, 1.000
No. of measured, independent and observed [*I* > 2σ(*I*)] reflections	10267, 1887, 1811	6924, 2189, 1949	11782, 2157, 2119
*R*_int_	0.016	0.028	0.020
(sin θ/λ)_max_ (Å^−1^)	0.617	0.616	0.616

Refinement			
*R*[*F*^2^ > 2σ(*F*^2^)], *wR*(*F*^2^), *S*	0.027, 0.077, 1.09	0.031, 0.089, 1.05	0.018, 0.048, 1.05
No. of reflections	1887	2189	2157
No. of parameters	123	136	137
No. of restraints	0	1	0
Δρ_max_, Δρ_min_ (e Å^−3^)	0.23, −0.25	0.24, −0.19	0.33, −0.30

**Table:** 

	**[DMPT(H)][HSO_4_]**	**[Dimer][BF_4_]_2_**	**[Dimer][HSO_4_]_2_·H_2_O**
Crystal data			
Chemical formula	C_9_H_14_N_2_O_4_S_2_^+^·HSO_4_^−^	C_18_H_24_N_4_S_2_^2+^·2BF_4_^−^	C_18_H_24_N_4_S_2_^2+^·2HSO_4_^−^·H_2_O
*M*_r_	278.34	534.15	572.68
Crystal system, space group	Monoclinic, *P*2_1_/*c*	Monoclinic, *C*2/*c*	Monoclinic, *P*2_1_/*c*
Temperature (K)	100	180	120
*a*, *b*, *c* (Å)	11.9033 (2), 13.3341 (3), 8.0321 (2)	15.6877 (2), 10.4883 (2), 14.8569 (2)	15.7786 (1), 10.5384 (1), 15.1791 (1)
β (°)	102.842 (2)	98.972 (1)	102.694 (1)
*V* (Å^3^)	1242.96 (5)	2414.60 (6)	2462.31 (3)
*Z*	4	4	4
μ (mm^−1^)	3.97	2.71	4.05
Crystal size (mm)	0.40 × 0.12 × 0.12	0.22 × 0.15 × 0.07	0.09 × 0.08 × 0.07

Data collection			
*T*_min_, *T*_max_	0.428, 1.000	0.732, 1.000	0.793, 1.000
No. of measured, independent and observed [*I* > 2σ(*I*)] reflections	11118, 2361, 2209	10154, 2347, 2180	25961, 4797, 4387
*R*_int_	0.044	0.020	0.039
(sin θ/λ)_max_ (Å^−1^)	0.615	0.616	0.617

Refinement			
*R*[*F*^2^ > 2σ(*F*^2^)], *wR*(*F*^2^), *S*	0.049, 0.127, 1.10	0.033, 0.089, 1.08	0.029, 0.088, 1.04
No. of reflections	2361	2347	4797
No. of parameters	176	168	361
No. of restraints	3	0	11
Δρ_max_, Δρ_min_ (e Å^−3^)	0.41, −0.72	0.31, −0.21	0.38, −0.42

Computer programs: *CrysAlis PRO* (Rigaku OD, 2025 ▸), *SHELXT* (Sheldrick, 2015*a* ▸), *SHELXL2018* (Sheldrick, 2015*b* ▸), *Mercury* (Macrae *et al.*, 2020 ▸) and *SHELXL* in *WinGX* (Farrugia, 2012 ▸).

**Table 2:** Selected hy­dro­gen-bond parameters (Å, °)

*D*—H⋯*A*	*D*—H	H⋯*A*	*D*⋯*A*	*D*—H⋯*A*
**DMPT**				
N1—H1N⋯S1^i^	0.856 (19)	2.464 (19)	3.2961 (12)	164.2 (15)
N2—H3N⋯S1^ii^	0.849 (17)	2.576 (18)	3.4183 (11)	171.6 (14)
				
**[DMPT(H)][Cl]**				
S1—H1*S*⋯Cl1^iii^	1.26 (2)	2.39 (2)	3.6064 (6)	162.4 (14)
N1—H1N⋯Cl1^iv^	0.87 (2)	2.33 (2)	3.1685 (15)	162.9 (19)
N1—H2N⋯Cl1^v^	0.94 (2)	2.42 (2)	3.2649 (15)	149.4 (19)
N2—H3N⋯Cl1^iii^	0.863 (9)	2.296 (10)	3.1467 (13)	168.7 (18)
				
**[DMPT(H)][Br]**				
S1—H1*S*⋯Br1	1.23 (2)	2.52 (2)	3.7176 (4)	162.9 (16)
N1—H1N⋯Br1^vi^	0.82 (2)	2.56 (2)	3.3363 (14)	160.1 (19)
N1—H2N⋯Br1^vii^	0.89 (2)	2.58 (2)	3.3725 (14)	148.4 (18)
N2—H3N⋯Br1	0.88 (2)	2.47 (2)	3.3298 (13)	165.4 (19)
				
**[DMPT(H)][HSO_4_]**				
S1—H1*S*⋯O3	1.18 (5)	2.54 (4)	3.645 (3)	154 (3)
S1—H1*S*⋯O4	1.18 (5)	2.60 (5)	3.678 (3)	150 (3)
N1—H1N⋯O3	0.874 (10)	1.935 (11)	2.807 (4)	175 (4)
N1—H2N⋯O4^viii^	0.877 (10)	1.985 (12)	2.859 (4)	174 (4)
N2—H3N⋯O1^viii^	0.878 (10)	1.968 (11)	2.845 (4)	178 (5)
O2—H2*S*⋯O1^ix^	0.84 (6)	1.79 (6)	2.627 (3)	173 (6)
				
**[Dimer][BF_4_]_2_**				
N1—H1N⋯F2^x^	0.91 (2)	1.90 (2)	2.8128 (17)	173 (2)
N1—H2N⋯F1^ii^	0.85 (2)	1.99 (2)	2.8105 (18)	161.3 (19)
N2—H3N⋯F4	0.84 (2)	2.02 (2)	2.8396 (18)	166 (2)
N2—H3N⋯S1^xi^	0.84 (2)	2.76 (2)	3.1502 (14)	110.1 (16)
				
**[Dimer][HSO_4_]_2_·H_2_O**				
N1—H1N⋯O1	0.877 (9)	1.951 (10)	2.8256 (16)	175 (2)
N1—H2N⋯O2^xii^	0.878 (10)	1.962 (11)	2.8172 (16)	164 (2)
N2—H3N⋯O3^xiii^	0.868 (9)	1.947 (10)	2.8125 (16)	175.2 (18)
N3—H4N⋯O7^xiv^	0.879 (9)	1.976 (10)	2.8440 (16)	169.3 (19)
N3—H5N⋯O8	0.877 (9)	2.132 (12)	2.9596 (16)	157.0 (19)
N4—H6N⋯O6^xv^	0.868 (9)	2.058 (12)	2.8703 (16)	155.5 (19)
O4—H1H⋯O1*W*	0.891 (10)	1.673 (11)	2.5571 (15)	171 (3)
O6—H2H⋯O3^xvi^	0.884 (10)	1.717 (10)	2.5966 (15)	173 (2)
O1*W*—H1*W*⋯O5^xvii^	0.865 (10)	2.36 (2)	2.9801 (16)	129 (2)
O1*W*—H2*W*⋯O8^xvii^	0.874 (10)	2.017 (13)	2.8515 (16)	159 (2)

Symmetry codes: (i) −*x* + 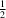, *y* − 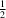, −*z* + 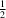; ii) −*x* + 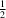, *y* + 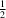, −*z* + 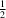; (iii) −*x* + 2, −*y*, −*z* + 1; (iv) *x* + 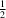, −*y* + 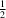, *z* + 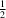; (v) −*x* + 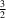, −*y* + 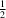, −*z* + 1; (vi) −*x* + 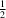, *y* + 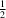, −*z* + 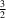; (vii) *x* + 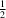, *y* + 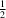, *z*; (viii) −*x* + 1, *y* − 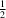, −*z* + 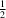; (ix) *x*, −*y* + 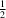, *z* + 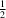; (x) *x*, −*y*, *z* − 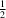; (xi) −*x* + 1, *y*, −*z* + 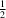; (xii) −*x*, −*y* + 1, −*z* + 1; (xiii) *x*, −*y* + 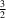, *z* + 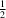; (xiv) −*x* + 1, −*y* + 1, −*z* + 2; (xv) −*x* + 1, *y* + 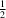, −*z* + 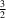; (xvi) −*x* + 1, *y* − 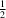, −*z* + 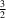; (xvii) −*x* + 1, −*y* + 1, −*z* + 1.

**Table 3:** Selected bond lengths (Å) for **DMPT** and pro­ton­ated **[DMPT(H)]^+^** species

**DMPT**			
S1—C1	1.6992 (12)	N2—C1	1.3447 (15)
N1—C1	1.3225 (16)	N2—C2	1.4429 (14)
			
**[DMPT(H)][Cl]**			
S1—C1	1.7530 (15)	N2—C1	1.312 (2)
N1—C1	1.308 (2)	N2—C2	1.4442 (18)
			
**[DMPT(H)][Br]**			
S1—C1	1.7508 (15)	N2—C1	1.318 (2)
N1—C1	1.313 (2)	N2—C2	1.4476 (19)
			
**[DMPT(H)][HSO_4_]**			
S1—C1	1.751 (3)	N2—C1	1.317 (4)
N1—C1	1.316 (4)	N2—C2	1.444 (4)

**Table 4:** Selected bond angles (°) and the dihedral angles (°) between the planes of thio­amide group and the C_6_ ring for the DMPT-containing structures

**DMPT**		**[DMPT(H)][Cl]**	
		C1—S1—H1*S*	94.5 (9)
C1—N2—C2	120.47 (10)	C1—N2—C2	122.45 (13)
N1—C1—N2	117.96 (11)	N1—C1—N2	122.01 (14)
N1—C1—S1	120.39 (9)	N1—C1—S1	117.69 (12)
N2—C1—S1	121.64 (9)	N2—C1—S1	120.29 (12)
Dihedral	80.82 (5)	Dihedral	89.94 (5)
			
**[DMPT(H)][Br]**		**[DMPT(H)][HSO_4_]**	
C1—S1—H1*S*	95.9 (11)	C1—S1—H1*S*	92 (2)
C1—N2—C2	122.33 (13)	C1—N2—C2	123.0 (3)
N1—C1—N2	121.70 (14)	N1—C1—N2	122.1 (3)
N1—C1—S1	117.21 (12)	N1—C1—S1	121.1 (2)
N2—C1—S1	121.08 (12)	N2—C1—S1	116.8 (2)
Dihedral	75.10 (4)	Dihedral	87.44 (10)

**Table 5:** Selected geometric parameters (Å, °) for the di­sulfide species

**[Dimer][BF_4_]_2_**			
N1—C1	1.305 (2)	C1—S1	1.7792 (15)
N2—C1	1.307 (2)	S1—S1^i^	2.0364 (8)
C1—S1—S1^i^—C1^i^	−95.78 (11)		
			
**[Dimer][HSO_4_]_2_·H_2_O**			
S1—C1	1.7879 (14)	N2—C1	1.3107 (18)
S1—S2	2.0431 (5)	N3—C10	1.3097 (19)
S2—C10	1.7804 (14)	N4—C10	1.3076 (18)
N1—C1	1.3106 (19)	C1—S1—S2—C10	97.20 (10)

Symmetry code: (i) −*x* + 1, *y*, −*z* + 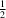.
