# Supplementary material for: Salt forms of a thio­amide: protonation of 1-(2,6-di­methyl­phen­yl)thio­urea
Source: Acta Crystallogr C Struct Chem. 2026 Feb 25;82(Pt 3):129–37. doi: 10.1107/S2053229626001804 (PMC12961746; doi:10.1107/S2053229626001804)
Supplement: Supplementary file 8 [file c-82-00129-sup8.pdf]

STRUCTURAL CHEMISTRY

Volume 82 (2026)

Supporting information for article:

Salt forms of a thioamide: protonation of 1-(2,6-dimethylphen-yl)thiourea

Kristin Donnachie, Bethany Gibson, Alan R. Kennedy, Connor MacCall and Marc Reid

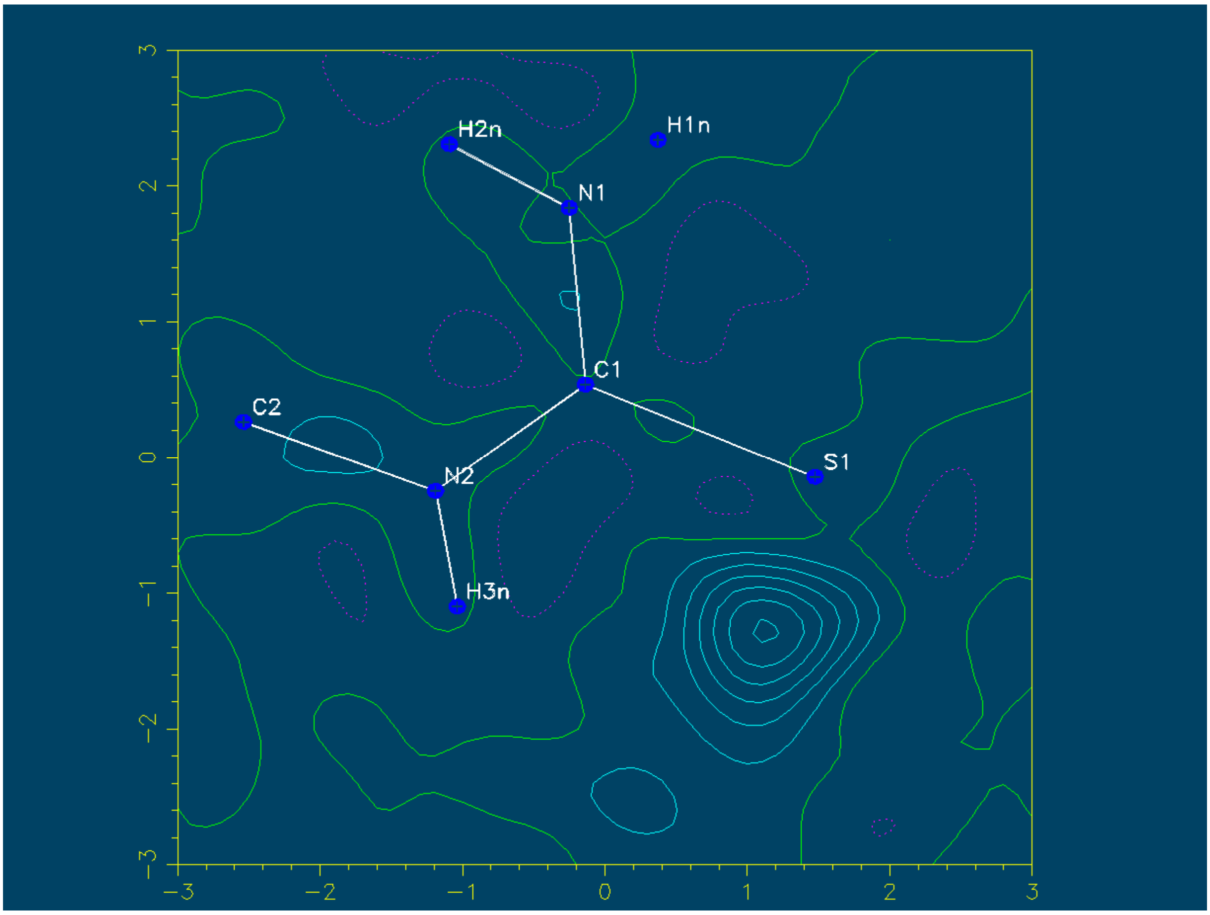

Supplementary Figure 1. Fo-Fc contour map showing the electron density peak next to S1 in the structure of **[DMPT(H)][Cl]** which was assigned as a proton in the final structure. Here, and Figures below, contour lines are each 0.1 e Å^{-3} and the x and y axes scales are in Å.

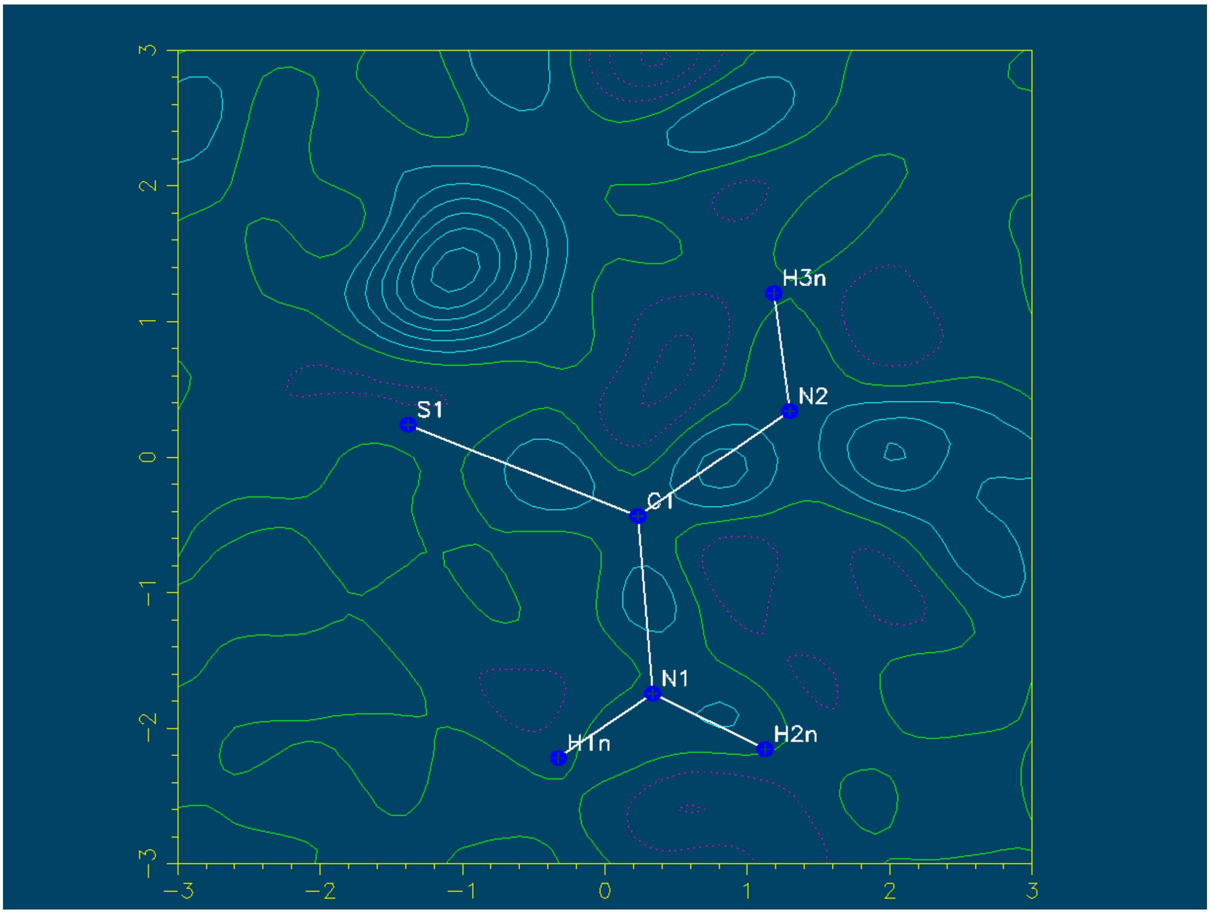

Supplementary Figure 2. Fo-Fc contour map showing the electron density peak next to S1 in the structure of **[DMPT(H)][Br]** which was assigned as a proton in the final structure.

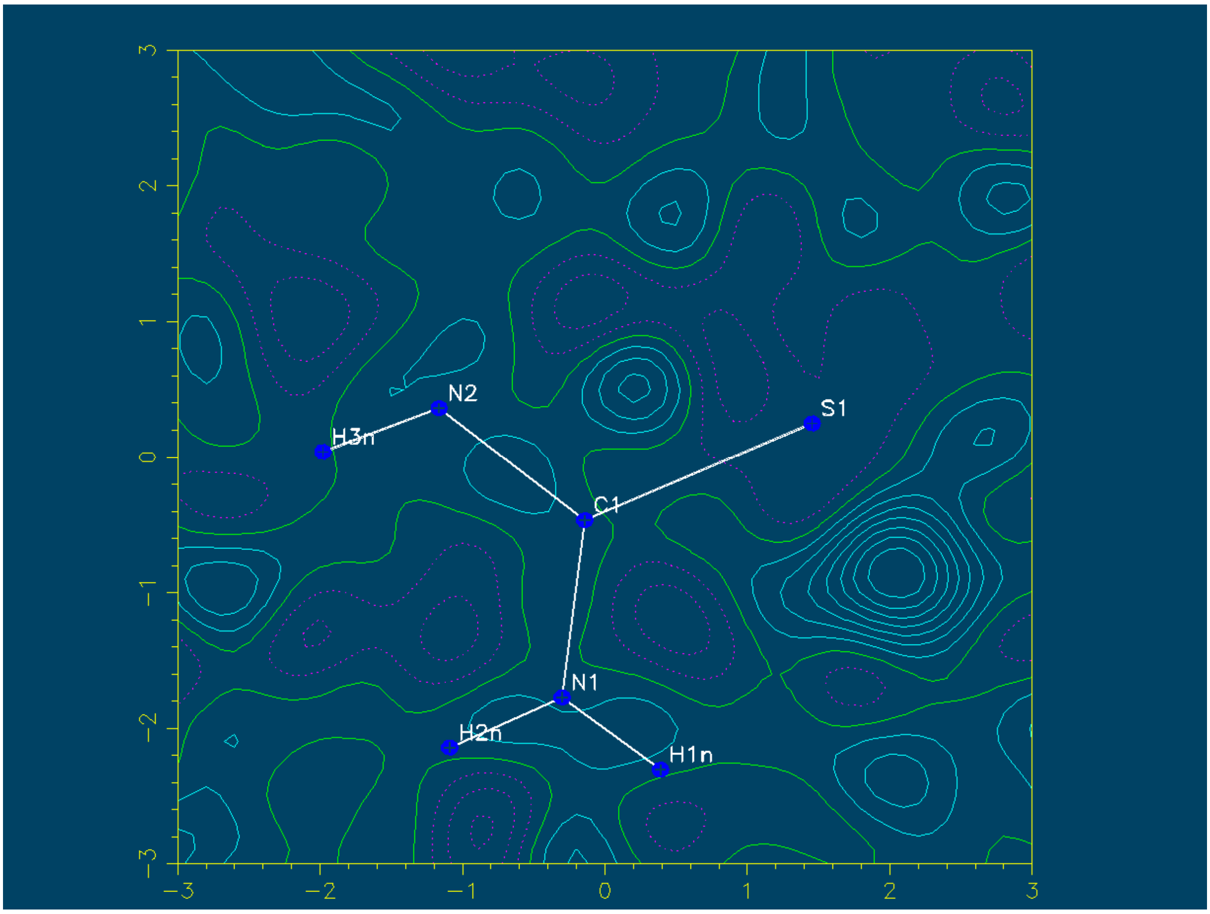

Supplementary Figure 3. Fo-Fc contour map showing the electron density peak next to S1 in the structure of **[DMPT(H)][SO_4H]** which was assigned as a proton in the final structure.
